# Mud Baths

**Published:** 1899-08

**Authors:** 


### MUD BATHS.

Volcano baths are the proper things nowadays in certain parts of California and Mexico. Down in Mendocino County, Cal., such baths have become most frequent.

The volcano bath is not a water bath, nor is it a fire bath nor a lava bath, as might be supposed. It is a mud bath, and no ordinary mud bath at that.

Ice-cold mud of a bluish tint and of the consistency of freshly mixed mortar is the element into which the bathers plunge, splashing and spluttering. The way they manage it is unique.

A sapling is felled in the forests near the volcano craters, stripped of its limbs, carried to the crater and placed across it, so that each end of the pole rests on firm ground.

Fancy yourself sliding out on one of these saplings stretched across a crater's mouth, then slipping gently off into the middle of a gurgling, bubbling, ice-cold mass of mud and swinging yourself there by your hands until fatigued! Then with just life enough left to crawl back along the log you reach unyielding ground again.

Fancy that situation and you may realize what volcano bathing is like. It is a combination of gymnastics and mud-dipping worth going miles to see. To hold onto the sapling is the thing, for to let go would mean horrible death. To let go of that pole would mean a visit to the interior of the earth—a sort of post-mortem Jules Verne exploration of a bottomless pit, as one writer puts it.

Once plunged into one of the craters of mud, with all the ties to the sapling severed, a person would be lost forever, being swallowed up in the murky depths in an instant, for vastly quicker in action and surer of its victim than quicksand is the mud of Mendocino's mysterious volcanoes.

Cleanliness has nothing to do with it. It is not that for which people face the dangers of the volcano bath. The mud which is belched forth from the earth's interior is supposed to contain important medicinal properties, and it is to be benefited by these that people risk their lives. Frail humanity will hesitate at nothing in search of health.

There are about twenty-five of these singular mud-belching volcanoes in Mendocino County, and they are among California's many wonders. They are situated high on a mountain side, seven miles from Cahto. At this time of the year they are unusually active. Their gurgling roar may be heard for a distance of several miles when they are most violent.

The mud frequently shoots over the rim of the crater, flows down the mountain like a lava stream and enters one of the Eel River's tributaries called Mud Creek. It fills the craters, which are about five feet above the earth's surface and bounded with a circular base or miniature crater from four to seven feet in diameter at the base and two to three feet at the top.

Prospecting parties have hewn down saplings fifty feet in length and pushed them into the mouth of a crater. Some of these have disappeared altogether. Others remain near the surface, playthings of the muddy element, which tosses them about like fishermen's bobbins in a rough sea.

A significant coincidence is the fact that when the ocean, twenty miles away, is unusually heavy and rough, the volcanoes become intensely active, belching forth not only their burden of ice-cold mud but volumes of warm vapor. In some mysterious way the ocean seems to control their action. —*Public Health Journal.*

